# Supplementary material for: Spatial Patterns of Anthrax Outbreaks and Cases among Livestock in Lesotho, 2005–2016
Source: Int J Environ Res Public Health. 2020 Oct 19;17(20):7584. doi: 10.3390/ijerph17207584 (PMC7588925; doi:10.3390/ijerph17207584)
Supplement: Supplementary file 1 [file ijerph-17-07584-s001.pdf]

## Annexure 1: Lesotho villages and their corresponding code

| ID | Village |
|---|---|
| 1 | Liqobong |
| 2 | Boiketsiso |
| 3 | Makhunoane |
| 4 | Qholaqhoe |
| 5 | Namahali |
| 6 | Maama |
| 7 | Tumane |
| 8 | Matlakeng |
| 9 | Chaba |
| 10 | Mopeli |
| 11 | Qalo |
| 12 | Khukhune |
| 13 | Nqechane |
| 14 | Butha-Buthe Urban |
| 15 | Sekubu |
| 16 | Tsime |
| 17 | Soloane |
| 18 | Linakeng (Butha Buthe) |
| 19 | Likhutlong |
| 20 | Leribe |
| 21 | Ha Mots'eare |
| 22 | Mohobollo |
| 23 | Pela Ts'oeu |
| 24 | Hlotse |
| 25 | Maputsoe |
| 26 | Pela-ts'oeu |
| 27 | Tsikoane |
| 28 | Likhakeng |
| 29 | Matlameng |
| 30 | Peka |
| 31 | Mahobong |
| 32 | Hleoheng |
| 33 | Mothae |
| 34 | Pitseng |
| 35 | Thaba Phats'oa (Leribe) |
| 36 | Lejone |
| 37 | Mokomahatsi |
| 38 | Liphakoeng (Butha Buthe) |
| 39 | Kolojane |
| 40 | Kolonyama |
| 41 | Fobane |
| 42 | Pae-Lea-Itlhatsoa |
| 43 | Molikaliko |
| 44 | Lipetu |
| 45 | Lephats'oane |
| 46 | Tlhanyaku |
| 47 | Bela-Bela |
| 48 | Mphorosane |
| 49 | Mokhachane |
| 50 | 'Mamathe |
| 51 | Seshote |
| 52 | Lehana-le-molapa |
| 53 | Majoe-Mats'o |
| 54 | Meno |
| 55 | Mapoteng |
| 56 | 'Makhoroana |
| 57 | Malotoaneng |
| 58 | Sebetia |
| 59 | Lekokoaneng |
| 60 | Mapholaneng |
| 61 | Suoane |
| 62 | 'Meta |
| 63 | Mohatlane |
| 64 | Motsitseng |
| 65 | Mongolo |
| 66 | Lebese |
| 67 | Mika |
| 68 | Koali |
| 69 | Maqhaka |
| 70 | Matsoku |
| 71 | Malingoaneng |
| 72 | Kosetabole |
| 73 | Moshemong |
| 74 | Popa (Mokhotlong) |
| 75 | Foso |
| 76 | Taung |
| 77 | Semenanyane |
| 78 | Thupa-Kubu |
| 79 | Mokhameleli |
| 80 | Khamolane |
| 81 | Thuathe |
| 82 | Tsoelike |
| 83 | Pulane |
| 84 | Mateanong |
| 85 | Berea Urban |
| 86 | Mokhotlong Urban |
| 87 | Maseru Urban |
| 88 | Maluba-Lube |
| 89 | Pontseng (Maseru) |
| 90 | Ratau |
| 91 | Thaba-Bosiu |
| 92 | Rafolatsane |
| 93 | Khokhoba |
| 94 | Liphakoeng (Mokhotlong) |
| 95 | Bokong |
| 96 | Sehong-hong |
| 97 | Setibing |
| 98 | Khohlontso |
| 99 | Bobete |
| 100 | Bobatsi |
| 101 | Mazenod |
| 102 | Makopoi |
| 103 | Popa (Maseru) |
| 104 | Moeketsane |
| 105 | Rothe |
| 106 | Linakeng (Mokhotlong) |
| 107 | Mantsonyane |
| 108 | Mofoka |
| 109 | Maboloka (Thaba Tseka) |
| 110 | Likalaneng |
| 111 | Roma |
| 112 | Lirahalibonoe |
| 113 | Nyakosoba |
| 114 | Thaba Moei |
| 115 | Linakeng (Thaba Tseka) |
| 116 | Manamaneng |
| 117 | Makhaleng |
| 118 | Lits'oeneng |
| 119 | Bokhoasa |
| 120 | Matsieng |
| 121 | Kolo |
| 122 | Mohlanapeng |
| 123 | Mochochoko |
| 124 | Mahlong |
| 125 | Setleketseng |
| 126 | Thaba-nts'o |
| 127 | Tsakholo |
| 128 | Serooeng |
| 129 | Mashai |
| 130 | Methalaneng |
| 131 | Rapoleboea |
| 132 | Litsoeneng |
| 133 | Tebang |
| 134 | Boleka |
| 135 | Morija |
| 136 | Thaba_Putsoa (Maseru) |
| 137 | Takalatsa |

| | |
|---|---|
| 138 | Moeaneng |
| 139 | Ramabanta |
| 140 | Tajane |
| 141 | Khutlo-Semetsi |
| 142 | Mathebe |
| 143 | Sehonghong |
| 144 | Sebelekoane |
| 145 | Sehlaba-Thebe |
| 146 | Thaba-Tsoeu (Mafeteng) |
| 147 | Ribaneng |
| 148 | Lesobeng |
| 149 | Semonkong |
| 150 | Qalabane |
| 151 | Likhoele |
| 152 | Matelile |
| 153 | Mphatsoenyane |
| 154 | Lebakeng |
| 155 | Malealea |
| 156 | Moeti |
| 157 | Malumeng |
| 158 | Mafeteng Urban |
| 159 | Matebeng |
| 160 | Ketane |
| 161 | Maboloka (Mafeteng) |
| 162 | Lekhatje |
| 163 | Qabane |
| 164 | Motsekuoa |
| 165 | Silooe |
| 166 | Mosenekeng |
| 167 | Ha Leronti |
| 168 | Thabana-Morena |
| 169 | Kokome |
| 170 | 'Melikane |
| 171 | Masemousu |
| 172 | Hloahloeng |
| 173 | Leseling |
| 174 | Tsatsa le Meno |
| 175 | Ha Thabo |
| 176 | Moshebi |
| 177 | Pontseng (Mohale's Hoek) |
| 178 | Linakeng (Qacha's Nek) |
| 179 | Mafikalisiu |
| 180 | Setofolo |
| 181 | Ha Thuube |
| 182 | Thaba-Chitja |
| 183 | Qhobong |
| 184 | Thaba-Tsoeu (Mohale's Hoek) |
| 185 | Mohlapiso |
| 186 | Likhutloaneng |
| 187 | Mamafi |
| 188 | Makhoareng |
| 189 | Tebellong |
| 190 | Matlali (Qacha's Nek) |
| 191 | Qaqatu |
| 192 | Ha Sekake |
| 193 | Mosuoe |
| 194 | Qhalasi |
| 195 | Rankakala |
| 196 | Qhoali |
| 197 | White Hill |
| 198 | Ha Teke |
| 199 | Pheellong |
| 200 | Sekhalabateng |
| 201 | Ts'ethe |
| 202 | Ha Kooko |
| 203 | Mount Austin |
| 204 | Mosaqane |
| 205 | Qalakheng |
| 206 | Qacha'snek Urban |
| 207 | Old Hoek |
| 208 | Dikolobeng |
| 209 | Lebekos |
| 210 | Phamong |
| 211 | Tlokotsi (Ha Masupha) |
| 212 | Ha Sehloho |
| 213 | Ha Mosehle |
| 214 | Matlali (Quthing) |
| 15 | Mohloka Kobo |
| 216 | Ha Ntsie |
| 217 | Ha Anone |
| 218 | Ha Peeta |
| 219 | Ha Roelane |
| 220 | Ha Mashapha |
| 221 | Ha Ratjeka |
| 222 | Morifi |
| 223 | Makaves (Ongeluk'snek) |
| 224 | Maphutseng |
| 225 | Putsoa-thaba (Dalvwa) |
| 226 | Ha Khakeng |
| 227 | Bodula thaba |
| 228 | Montsi |
| 229 | Ha Maqalo |
| 30 | Mekaling |
| 231 | Ha Mohlakoana |
| 232 | Sebapala |
| 233 | Mount Moorosi |
| 234 | Ha Trans (Ha Liphapang) |
| 235 | Ha Tsui |
| 236 | Nene |
| 237 | Ha Malephane |
| 238 | Ha Hlapa |
| 239 | Sempe |
| 240 | Tele-Tele |
| 241 | Moyeni |
| 242 | Ha Phoka |
| 243 | Lehana Pass |
| 244 | Moyeni Camp |
| 245 | Mtjanyane (east) |
| 246 | Mtjanyane (west) |
| 247 | Quthing Urban |
| 248 | Matsatseng |
| 249 | Sdakeni |
| 250 | Bolepeletsa |
| 251 | Tosing |
| 252 | Fort Hartley |
| 253 | Dili-Dili |
| 254 | Nthodimonate |
| 255 | Makhetheng |
| 256 | Ha Mpeka |
| 257 | Ha Mokhethoane |
| 258 | Mphojoa |
| 259 | Sinxondo |
| 260 | Tele |
